# Visual-spatial processing impairment in the occipital-frontal connectivity network at early stages of Alzheimer’s disease

**DOI:** 10.3389/fnagi.2023.1097577

**Published:** 2023-02-09

**Authors:** Iván Plaza-Rosales, Enzo Brunetti, Rodrigo Montefusco-Siegmund, Samuel Madariaga, Rodrigo Hafelin, Daniela P. Ponce, María Isabel Behrens, Pedro E. Maldonado, Andrea Paula-Lima

**Affiliations:** ^1^Department of Medical Technology, Faculty of Medicine, Universidad de Chile, Santiago, Chile; ^2^Biomedical Neuroscience Institute, Faculty of Medicine, Universidad de Chile, Santiago, Chile; ^3^Institute of Neurosurgery and Brain Research Dr. Alfonso Asenjo, Santiago, Chile; ^4^Department of Neuroscience, Faculty of Medicine, Universidad de Chile, Santiago, Chile; ^5^Faculty of Medicine, Institute of Locomotor System and Rehabilitation, Universidad Austral de Chile, Valdivia, Chile; ^6^Department of Neurology and Neurosurgery, Hospital Clínico Universidad de Chile, Santiago, Chile; ^7^Faculty of Medicine, Center for Advanced Clinical Research, Universidad de Chile, Santiago, Chile; ^8^Department of Neurology and Psychiatry, Clínica Alemana-Universidad del Desarrollo, Santiago, Chile; ^9^Institute for Research in Dental Sciences, Faculty of Dentistry, Universidad de Chile, Santiago, Chile

**Keywords:** mild cognitive impairment (MCI), spatial memory, virtual navigation, EEG, eye-tracking, Alzheimer’s disease

## Abstract

**Introduction:**

Alzheimer’s disease (AD) is the leading cause of dementia worldwide, but its pathophysiological phenomena are not fully elucidated. Many neurophysiological markers have been suggested to identify early cognitive impairments of AD. However, the diagnosis of this disease remains a challenge for specialists. In the present cross-sectional study, our objective was to evaluate the manifestations and mechanisms underlying visual-spatial deficits at the early stages of AD.

**Methods:**

We combined behavioral, electroencephalography (EEG), and eye movement recordings during the performance of a spatial navigation task (a virtual version of the Morris Water Maze adapted to humans). Participants (69–88 years old) with amnesic mild cognitive impairment–Clinical Dementia Rating scale (aMCI–CDR 0.5) were selected as probable early AD (eAD) by a neurologist specialized in dementia. All patients included in this study were evaluated at the CDR 0.5 stage but progressed to probable AD during clinical follow-up. An equal number of matching healthy controls (HCs) were evaluated while performing the navigation task. Data were collected at the Department of Neurology of the Clinical Hospital of the Universidad de Chile and the Department of Neuroscience of the Faculty of Universidad de Chile.

**Results:**

Participants with aMCI preceding AD (eAD) showed impaired spatial learning and their visual exploration differed from the control group. eAD group did not clearly prefer regions of interest that could guide solving the task, while controls did. The eAD group showed decreased visual occipital evoked potentials associated with eye fixations, recorded at occipital electrodes. They also showed an alteration of the spatial spread of activity to parietal and frontal regions at the end of the task. The control group presented marked occipital activity in the beta band (15–20 Hz) at early visual processing time. The eAD group showed a reduction in beta band functional connectivity in the prefrontal cortices reflecting poor planning of navigation strategies.

**Discussion:**

We found that EEG signals combined with visual-spatial navigation analysis, yielded early and specific features that may underlie the basis for understanding the loss of functional connectivity in AD. Still, our results are clinically promising for early diagnosis required to improve quality of life and decrease healthcare costs.

## Introduction

Alzheimer’s disease (AD) is the primary cause of dementia and one of the leading sources of social and economic impact worldwide (Tahami Monfared et al., 2022). However, the pathophysiology and mechanisms related to some of the major clinical manifestations of AD remain elusive, as does the development of practical biomarkers to apply at early stages in the course of the disease. AD generates alterations in neuronal connectivity at early stages by damaging neuronal circuits and creating aberrant networks (Sarter and Bruno, 2004), contributing to memory loss and higher-order cognitive dysfunctions due to the neural circuits’ disconnection (Dauwels et al., 2010, 2011). Spatial memory loss represents one of the earliest signs of its clinical syndrome (Klimkowicz-Mrowiec et al., 2008; Hamilton et al., 2009; Etchamendy et al., 2012). It has been proposed that AD patients generate, at very early stages, detectable physiological changes during memory encoding processing (Bangen et al., 2012). The core network for navigation is constituted by the hippocampus, parietal, prefrontal, and occipital regions, which are activated in young and elderly controls, but not in AD and Mild Cognitive Impairment (MCI) subjects. This network impairment affects several frontal lobe areas of action planning sequences, including executive functioning, organization, error monitoring, and global response decision-making (Vann et al., 2009; Coughlan et al., 2018; Sneider et al., 2018).

We aimed to identify early behavioral and cerebral activity signs of AD by assessing electroencephalographic features recorded during the performance of patients at early AD (eAD) stages in a hippocampus-dependent, virtual spatial memory task [the Virtual Morris Water Navigation (VMWN)]. We conjectured that fixation-event-related potentials (fERPs) would reflect alterations in the sensory coding needed to perform this task successfully. We found that electroencephalography (EEG) signals, combined with visual-spatial navigation analysis, yielded early and specific features. These characteristics are not only the basis for understanding the loss of functional connectivity in AD; they are also clinically promising for early diagnosis and fall in line with the attributes suggested by health organizations, to improve patient’s quality of life and avoid significant healthcare costs (Cummings et al., 2013; Alberdi et al., 2016; Jack et al., 2018).

## Materials and methods

### Participants

The Scientific and Ethics Committee approved all procedures involving participants of the Clinical Hospital of the Universidad de Chile, Protocol number: 26/2015. A total of 38 individuals were initially recruited for this study. All participants signed informed consent. Participants were classified by a dementia-specialized neurologist blind to the participant’s performance in the navigation task. A total of 18 individuals, 9 at the early phase of AD and 9 cognitively healthy controls (HCs) aged 61–88 years, considering a strict criterion of EEG signal quality, were included. A total of 18 participants (11 from the control and 7 from the eAD group) were excluded because of artifactual or technical problems with the EEG recordings, due to the lack of posterior clinical confirmation of AD, or finally, to have an age-matched control group. The exclusion criteria for the eAD group were evidence of non-degenerative dementia (e.g., inflammatory, metabolic, or vascular dementia), non-amnestic MCI or cognitive impairment of doubtful origin, or severe medical conditions that limited their ability to participate in the study. Only the records of those patients who progressed to AD clinical diagnosis were included in the analyzes presented in this study. Control participants underwent the same neurological and neuropsychological evaluations as the eAD group. The demographic data are shown in Supplementary Table 1.

### Neuropsychological testing

Subject cognitive status was evaluated with the Clinical Dementia Rating scale (CDR) (Morris, 1993), CDR Sum-of-Boxes (CDR-SOB) (O’Bryant et al., 2008), the mini-mental state examination (MMSE) (Llamas-Velasco et al., 2015), the Montreal Cognitive Assessment (MoCA) (Nasreddine et al., 2005) validated in our country (Delgado et al., 2019), and the MoCA Memory Index Score (MoCA-MIS) (Julayanont et al., 2014). The maximum score of the MoCA test is 30; a score equal to or greater than 26 is considered normal according to normed and validated scales for its administration; however, the cutoff was lower (<21) in the validation in our population (Delgado et al., 2019). The early stage AD group, hereafter designated as eAD, consisted of patients with cognitive impairment with memory deficits confirmed by an informant. The global CDR in all eAD participants was 0.5; the CDR-SOB was ≥ 1.5, and the MoCA-MIS ≤ 11, with two or fewer out of five words recalled spontaneously. All eAD participants had a very mild loss of instrumental activities in daily living but did not comply with the diagnosis of dementia.

### Behavioral task: Virtual Morris Water Navigation (VMWN) task

Spatial exploration by the eAD and control participants was assessed with the VMWN task, implemented through an open-use program adjusted under the same parameters described in (Hamilton et al., 2009), and with support provided by its author. The virtual environment simulates the traditional Morris water maze navigation task, consisting of a circular pool inside a room with visual cues on its four walls. By convention in the study, measurements are described in arbitrary units. The room measured 16 units wide and length by 3 units high, while the visual cues measured 3 × 3 units. The circular pool was 3.2 units in diameter, with a perimeter wall of 0.66 units above the pool surface. The square platform was 0.66 units wide and long and extended approximately 0.33 units above the pool surface. This description is the same for both the training and the task, where the only difference is the content of the visual cues (a figure of distinctive design). The virtual task is presented through a computer screen that the user must navigate by pressing keyboard buttons to find a platform hidden under the water. After finishing the task, the program stores the information about the route traveled in the text. The navigation task was divided into three stages. Stage I (Training): this task consisted of finding the hidden underwater platform in a room with visual clues on each of its walls, starting each repetition from a different location. Participants had to reach the platform and had 2 s to turn on the spot and visually explore their position in the environment. The platform became visible after 1 min if the participants did not find it. This training task comprised four trials, keeping the platform in the same hidden position inside the pool. Stage II (Task I): the participants had to find the hidden platform in the same pool based on a different set of visual cues. As in the previous stage, the platform became visible if not found after 1 min. The task comprised 20 trials divided into 4 blocks, allowing rest and eye-tracking recalibration. The platform’s location was the same throughout the task, and participants started each repetition from different positions. Stage III (Task II): in an equivalent virtual room with other visual cues, participants had to select between two visible platforms; only one represented the correct option. This task also consisted of 20 trials divided into 4 groups. Both platforms always maintained the same position. The participants started each repetition from a different location in the pool. We used this task to control each participant’s visual and psychomotor functioning, making it possible to rule out any deterioration in these parameters as possible explanations for faulty performance in Task I.

### EEG recordings and eye-tracking

An EEG system of 32 + 8 channels was used (32 EEG channels, 8 external channels to measure electro-ocular activity, and for referential mastoid recording; BioSemi^®^).^1^ The EEG electrodes were positioned with an elastic cap over the head. EEG activity was continuously acquired during the tasks. The acquired analog signal was filtered between 0 [real direct current (DC)] and 1,000 Hz, sampled at 2,048 Hz, and digitally converted with a precision of 24 bits. The recording system used a specific reference system Common Mode Sense (CMS) active electrode and Driven Right Leg (DRL) (CMS/DRL) that allows information storage. Each participant was placed in a comfortable seat in front of the monitor where the VMWN task was deployed. Their heads were positioned with chin support to minimize movement during the task and to allow optimal detection and recording of eye movements. Eye-tracking was accomplished by employing an Eyelink^®^ 1000 system, which digitizes and stores eye-tracking data in a binary file convertible to text, from which the bi-dimensional position of the pupils was obtained at a frequency of 500 Hz. The system automatically recorded blinks, fixations, and saccades based on user-defined initial parameters. The proximity to the platform was calculated by the software as a rank-sum test for all pixels of the heat map.

### Data analysis

All data analyses, including behavioral parameters of space navigation in the VMWN task, electroencephalographic signals, and eye-tracking data, were performed with MATLAB^®^ (The MathWorks, Inc., Natick, MA, USA). The data parsing pipeline was the same for the eAD and control groups. Text files of behavioral tasks were imported and analyzed using custom algorithms. Binary EEG files were imported and preprocessed using the Fieldtrip open-source toolbox. The EYE-EEG MATLAB toolbox was used as a plugin of the EEGLAB package to import, visualize, and verify the detected eye-tracking events and synchronize them with the EEG signals (Delorme and Makeig, 2004).

#### Sample sizing and analyzed data

We used convenience sampling and recruited data from 38 participants classified by a specialist into control and AD groups under neuropsychological testing and fulfilling different inclusion criteria. Subjects who did not have a recording of adequate quality for EEG analysis were ruled out, including the loss of electrodes necessary for the study and artifacts that significantly affect the signal. Eighteen subjects, nine control, and nine AD subjects, were then analyzed.

For the behavioral data, each subject contributed 20 trials to the analysis. For the ocular data, for each subject, the total number of fixations, and saccades for each trial was calculated for each subject. For electrophysiological analyses, fERP, time-frequency (TF) decomposition, and coherence, nearly 300 epochs for each participant were obtained. Each epoch corresponds to one eye fixation. The initial EEG data matrix was, therefore, made up of a *M* × *N* × *O* matrix, where the dimension *M* is given by the EEG channels (Perrochon and Kemoun, 2014), *N* by the time points sampled for each epoch of ocular fixation (1,500), and *O* by the number of epochs of eye fixation throughout the 20 trials of the task (typically close to 3,000 epochs of eye fixation). The eye movement matrix, in turn, consisted of an *M* × *N* matrix for each eye, where *M* is the time points sampled during the task (1,200,000 points), *N* is the position variables on the *X*-axis, on the *Y*-axis, and acceleration of eye movement. Finally, the motor behavior matrix was made up of an *M* × *N* matrix, where *M* is the number of temporary samples taken throughout the task (1,200,000 points), and *N* is the position variables on the *X*-axis and position on the *Y*-axis of the navigation environment. Therefore, each of these million-point matrices has been sampled by each subject for the entire set of subjects in each analysis group.

#### Behavioral analysis

The parameters quantified were: (i) error rate corresponding to the fraction of trials where the participants could not find the platform within the first 60-s, (ii) latency in finding the platform (total time traveled); (iii) travel speed; (iv) length of the route followed to reach the platform; (v) resting time, defined as the total period in which participants visually explore the maze environment without moving; and (vi) latency after the platform became visible.

#### Eye-tracking analysis

We studied ocular parameters in terms of the number, duration, and frequency across trials. Fixations and saccades were automatically identified based on the velocity (30°/s) and acceleration threshold (8,000°/s2). Saccades longer than 5 ms and smaller than 100 ms and fixations between 50 and 800 ms were picked for further processing. In addition, blinks were defined as the absence of pupil data.

#### EEG analysis

Electroencephalography signals were inspected by a clinical neurophysiologist to evaluate the acquired signals’ quality. This procedure allowed the discard of abnormal recordings, including epileptiform activity and abnormal basal rhythms. Then the segments of data were marked for exclusion from the analysis. Offline filter settings were bandpass filter at 1–40 Hz (Butterworth, FIR). Next, artifact elimination was applied using the independent component analyses (ICA) decomposition (fieldtrip toolbox) over the continuous record. Finally, noise components were semi-automated, utilizing algorithms executed in the EYE-EEG package to detect ocular movement-related components.

Eye-tracking signals synchronized with segments of the continuous EEG signal, between −1,000 and 3,000 ms around the ocular fixations, were used. For each trial, we evaluated the first 30 s of recording to avoid variability in each participant’s time. Fixation-related epochs were subsequently subjected to analyses in the time and frequency domains: (1) fERPs were computed. For the fERP analysis, a reference correction was calculated by removing the baseline difference between −200 to 0 ms before eye-fixation (onset time). The resulting fERP between groups was compared in terms of amplitude, latency, phase, and scalp distribution, (2) the TF decomposition on the EEG epochs tapered by a sliding Hanning window, using a fixed window length for the frequency range from 1 to 40 Hz in 1 Hz steps between −750 and 1,500 ms, was computed. (3) We implemented a multitaper analysis based on multiplication in the frequency domain, obtaining output power spectra. (4) For each frequency, values of power-spectra were transformed to *Z* scores, normalizing by the corresponding mean and standard deviation (SD) of pre-stimulus time between −750 and −450 ms. (5) The normalized values for each time, frequency, and electrode between groups were compared. (6) For the amplitude of fERP and the TF charts between groups, a region of interest was defined as O1-Oz-O2 electrodes as the place of maximal amplitude for visual stimuli.

Also, we computed the coherence by performing TF analysis on any time series trial data using the multitaper method, based on conventional Hanning tapers, to obtain the power and the cross-spectral (fieldtrip toolbox). The estimated coherence ranges from 0 to 1, where 0 means that both signals are linearly independent, and 1 means that the frequency components of the signals have a maximum linear correlation. Thus, coherence estimation is a valuable tool for observing and quantifying the synchrony property of two EEG series, mainly when they are limited to particular frequency bands (Dauwels et al., 2010). Coherences for delta (2–4 Hz), theta (4–7 Hz), alpha (7–13 Hz), beta (13–30 Hz), and gamma bands (30–40 Hz) as the mean coherence values of the epochs between 0 and 300 ms, were calculated. The EEG coherence calculation for each electrode pair generates a 14 × 14 (channels selected) matrix showing the connectivity between all possible functional independent brain areas in each frequency band. To estimate whether the changes were primarily related to alteration of short- or long-range coherences reported to be affected in AD progression, the following electrodes were used: (O1, O2, PO3, PO4, CP1, CP2, C3, C4, F3, F4, F7, F8, Fp1, Fp2). Finally, we measured the effect sizes of the beta coherence spectrum (Cohen’s *d*) between groups.

### Statistical analyses

Demographics and neuropsychological performance were divided into continuous variables expressed as mean and SD, while the categorical variables were expressed as frequencies (%). Wilcoxon rank-sum test was used for age, education, CDR-SOB, MoCA, MoCA-MIS, and MMSE comparison between groups. A chi-square test was used for gender comparison (Fisher’s exact test). Differences in ocular behavior as the frequency of ocular movements (fixations and saccades), were measured using a Wilcoxon rank-sum test between groups. Additionally, we obtained a heat map of the probability of differences and a map of significant differences between the exploration performed by both groups. Finally, we performed a rank-sum test as a statistical approach pixel by pixel on the image at the 0.05 significance level.

Statistical tests based on permutations were applied to evaluate the differences in the oscillatory activity between groups. The Montecarlo method was considered an estimator of the permutation’s significance probabilities (two-tailed, alpha: *p* < 0.01, cluster correction, cluster-alpha: *p* < 0.05). Given many comparisons applied by the number of electrodes between groups, we used cluster as a correction method that solves the Multiple Comparison Problem (MCP) (Maris et al., 2007). Moreover, we calculated effect sizes (Cohen’s *d*) as the difference of the means of groups divided by the weighted pooled SDs of the groups. Cohen’s *d* effect size of 0.2–0.3 is a “small” effect, around 0.5 a “medium” effect, and from 0.8 to infinity, a “large” effect. We estimated significant differences in intragroup for the baseline in a beta-band coherence region of interest (15–20 Hz and between 0 and 300 ms) using a statistical threshold criterion of effect size (>0.5) and one-sample *t*-test (<0.05). A chi-square test for intragroup ratios was applied to this threshold. We processed the data with MATLAB and Fieldtrip toolbox.

## Results

The detailed demographic and neuropsychological assessment information is presented in Supplementary Table 1. Nine patients diagnosed with eAD, and nine matched control participants were included in this analysis. We found no significant differences in age, sex subgroups, and years of education. Neither were differences in the prevalence of diabetes, hypertension, or tobacco use between groups. In contrast, the MoCA and MoCA-MIS scores of the eAD group were significantly different from the control group (see Supplementary Table 1).

### Spatial navigation performance

**FIGURE 1:**
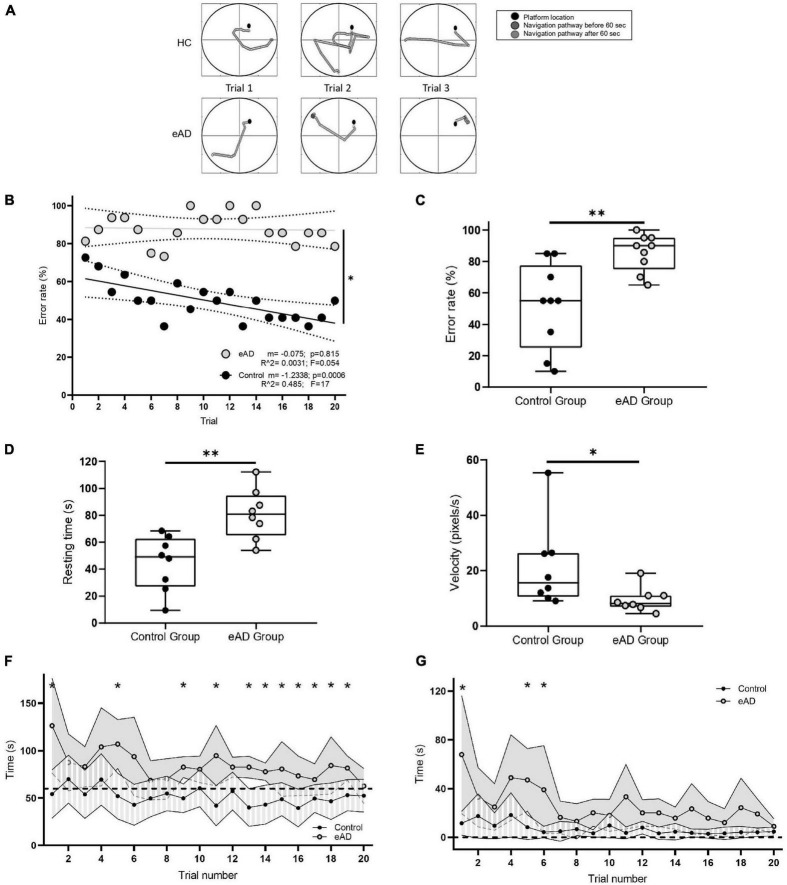
Navigation performance. **(A)** Examples of navigation paths to find the platform during the performance of the Virtual Morris Water Navigation (VMWN) memory task for the early Alzheimer’s disease (eAD) and the healthy control (HC) groups in three different trials. Trajectories when the platform was hidden (dark gray) and when the platform became visible after 60 s of navigation (light gray). **(B)** Multiple learning linear regression analysis for the control and eAD groups. The decrease in the error rate was significantly different between both groups [multiple lineal regression (MLR), *F* (1, 36) = 7.072, *p* = 0.0116]. **(C)** Error rate. Percentage of participants who could not find the platform. For panels **(C–E)**, the box-whisker plot represents the median error rate of the nine participants in the 20 trials for each group, and the statistical significance, determined by the Mann–Whitney test (*p < 0.05, ^**^p < 0.01). **(D)** Average resting time in which the participants in each group were not performing movements. **(E)** The speed at which the participants moved to the supposed position of the hidden platform. **(F)** Time spent to reach the platform in each of the 20 trials. Dashed line: 60 s–limit to fail (Wilcoxon rank-sum test, *p < 0.05). **(G)** Time spent by the participants to reach the platform after it became visible. Dashed line: time 0–the moment when the platform becomes visible (60 s after the initiation of the original trial). Points joined by the continuous line represent the mean per group, and for each group, the variance is also plotted. The eAD group showed a latency greater than the HC at the beginning of the test (Sidak corrected, *p < 0.05). **(B–F)** Gray: eAD participants (*n* = 9); black: HC participants (*n* = 9).

The experimental protocol and the setup are illustrated in Supplementary Figure 1. Representative examples of the trajectories taken by the eAD and the control participants in the VMWN are shown in Figure 1A. Control participants typically learned to find the hidden platform within the first 60 s of the task across trials. The error rate was significantly higher in eAD patients when compared to controls (Wilcoxon Rank sum test, *p* < 0.001) both across trials (Figure 1B) and between the groups along all the trials (Figure 1C). The eAD group displayed higher resting times than the control group. These results may indicate that eAD participants may need more time for visual processing and orientation before moving their position within the maze (Figure 1D). Accordingly, navigation speed was consequently reduced in the eAD group (Figure 1E). The average latency to find the platform also showed differences. For the eAD group, the average latency exceeded 60 s in most trials, indicating that these participants did not encounter the platform before it became visible (Figure 1F). Thus, in the case of the control group, participants found the hidden platform after five trials, while the AD group could never achieve the goal during the 60 s of the task, but they could find the platform once it became visible. Subsequent analyses based on this background consider similar exploratory behavior times for both groups, i.e., the first 30 s. This result suggests that most of the latency displayed by the eAD participants was mainly due to hesitation to execute the navigation task. After the platform became visible at 60 s, significant differences in latency were found only in the first trials, which were no longer significant after the sixth trial (Figure 1G). This result suggests that both groups could adequately execute the basic motor programs necessary for navigation, albeit with a slower improvement over time in the eAD group. Therefore, we can infer that the difficulty in carrying out the initial navigation task in the eAD group was not due to changes in the motor spectrum abilities.

### Ocular movement parameters and visual exploring strategies

To explore whether differences in spatial learning could be explained by changes in the ocular behavior of the groups, we compared the number and duration of fixations and saccades made during the task. Given the variation in reaching the platform over time, we calculated the total number of occurrences and their duration in the first 30 s. For fixations and saccades, participants showed no significant differences between groups in frequency and duration, indicating that the differences did not stem from alterations in basic ocular parameters. However, higher dispersion was observed in the eAD group (Supplementary Figure 2).

**FIGURE 2:**
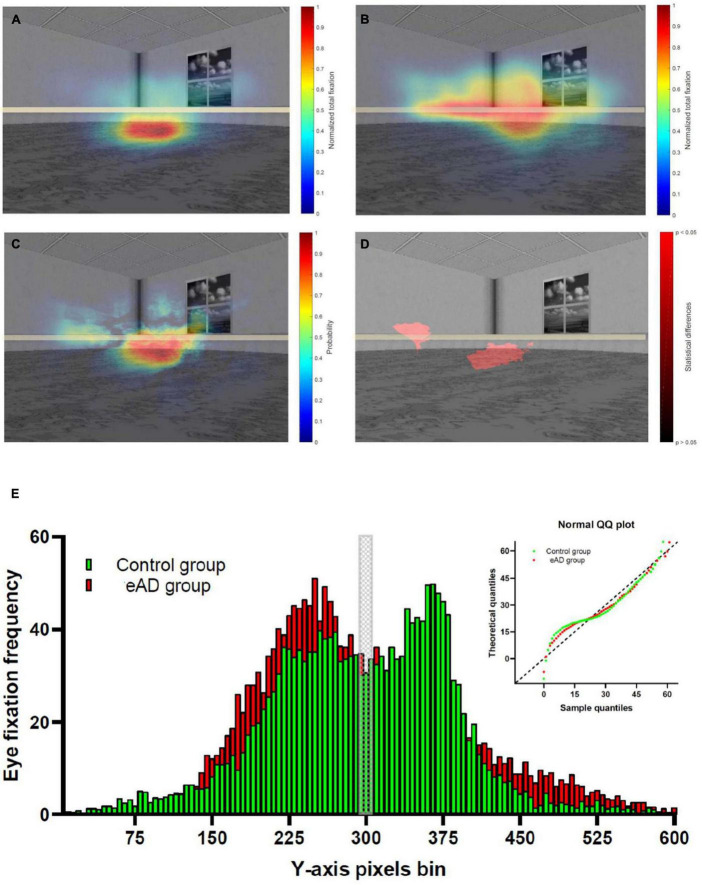
Heat map of the spatial distribution of eye-fixation of the control and early Alzheimer’s disease (eAD) group. **(A)** Heat map of eye-fixation of the control group, with high fixation density in the center. **(B)** Heat map of eye fixation of the eAD group demonstrated a less localized exploration. Warmer colors represent a higher number of fixations. **(C)** Heat map of the probability differences in the visual exploration. Warmer colors represent a higher probability and in panel **(D)** regions with statistical differences in visual exploration between control and eAD group. The red path represents statistical differences (Wilcoxon rank-sum test, *p* < 0.05). The horizontal gray line represents the central region of the image. **(E)** Comparison eye fixation frequency distribution in *Y*-axis pixels bin between control and eAD group, Kolmogorov–Smirnov test comparison cumulative fixation fraction (K–S test; *D* = 0.11, *p* = 0.0014). The gray block represents the central region of the image, while control group and eAD group results are represented in green and red, respectively.

We obtained a graphical representation of the distributions of ocular fixations by group using heat maps. Eye fixations in the control group were focused on a central region located at the representation of the water where the platform should be hidden (Figure 2A). Instead, in the eAD group, visual scanning was much more heterogeneous, with no clear preference (Figure 2B). We calculated absolute differences in the visual exploration between the groups, finding that although both groups visually scanned the center of the image in contrast to the periphery, the main differences were observed in the intermediate image region. Notably, the control group preferred to fix the central area, where the platform could be placed. In contrast, the eAD group focused on the upper midline without a defined focus on the target platform (Figure 2C).

In addition, we performed a pixel-by-pixel comparison of the visual scan of the groups. We used level curves for the eye fixation heat maps with the normalized values. Furthermore, we applied a rank-sum test for all pixels of the heat map to obtain a probability of differences. This result highlights the lower central region with statistical differences, which could correspond to an area close to the hidden platform (Figure 2D). We analyzed the distribution to confirm that the visual scanning behavior of participants with eAD differs from controls who fixate more on a specific position. Eye fixation frequency distribution revealed a significant difference between groups (K–S test; *D* = 0.11, *p* = 0.001). The eAD group had more eye fixations over the middle line relative to controls (Figure 2E). These results confirm that both groups differ behaviorally in their visual exploration, reflecting their ability to process visual information.

### Fixation event-related potentials (fERPs)

We explored early neuronal responses of the visual cortex elicited during each visual fixation performed along the task. This ERP reflects the visual processing that is putatively needed to properly extract the visual characteristics of the environment to complete the task. Thus, it was chosen as an integrity marker of cortical signals to differentiate between the eAD and control groups. fERP signals were computed as the average activity at the onset of fixations during the first 30 s of each trial (with 800–1,200 occurrences across the 20 trials).

**FIGURE 3:**
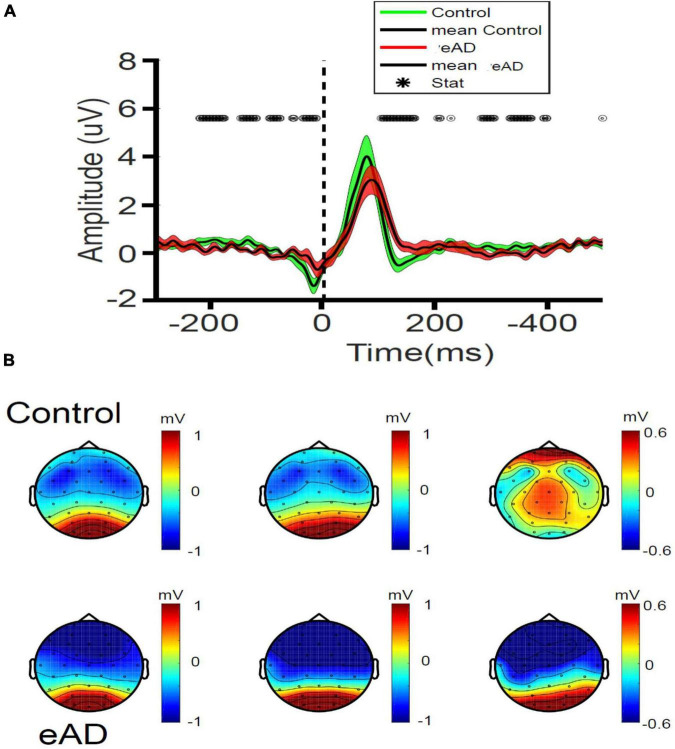
Fixation related potentials (RPs) recorded in early Alzheimer’s disease (eAD) and control participants. **(A)** Amplitude of the occipital potentials (mean of O1, O2, and Oz channels). The central black line represents the mean, and the colored shade the variance. For each curve, time 0 represents the onset of visual fixation. Statistical differences in time are indicated as black open circles at the top (Wilcoxon test, *p < 0.05). For panels **(A,B)**, red represents eAD patients (*n* = 9); and green, elderly controls (*n* = 9). **(B)** Scalp topographic maps of fixational-event RPs (fERPs) recorded at 60, 100, and 135 ms post-fixation. 2-D spatial color maps of voltage scalp distributions of fERP (in μV), from negative (blue) to positive voltages (red), at different latencies after the onset of fixation, are shown. Almost no spreading of the occipital dipole to parietal or frontoparietal regions is observed at later latencies in the eAD group compared to the control group.

We found a lower amplitude of occipital fERP signals in eAD participants compared to the control group, as shown in Figure 3A. Significant differences were observed in the period that preceded the fixation onset, including but not limited to the preceding saccade, meaning that the influence of the prior motor behavior was differentially observed in both groups. Another significant difference was observed between 0 and 200 ms from the onset of visual fixations, demonstrating that the early visual activity during each visual fixation displays a larger magnitude in controls than in eAD participants. We also found differences in the amplitude of potentials after the P100 component. Because brain signals have reached extra striate association cortices at these latencies, we examined the scalp distribution of fixational evoked potentials at different latencies to determine whether there were differences in the propagation of the electrical dipole (Figure 3B). In the control group, we observed an early activation of the occipital cortex compatible with the arrival of the visual input at 60 ms, then a maximum response near 100 ms, and finally, the frontal propagation occurring at about 135 ms. Remarkably, there was a shallow spreading of occipital activity to parietal and frontoparietal regions at later times in the eAD group compared to that observed in the control group, with a clear occipitofrontal dipole. These spatial differences suggest that visual processing differs between eAD and HCs at early visual cortices and higher spatial association cortical areas.

### Time-frequency analysis

**FIGURE 4:**
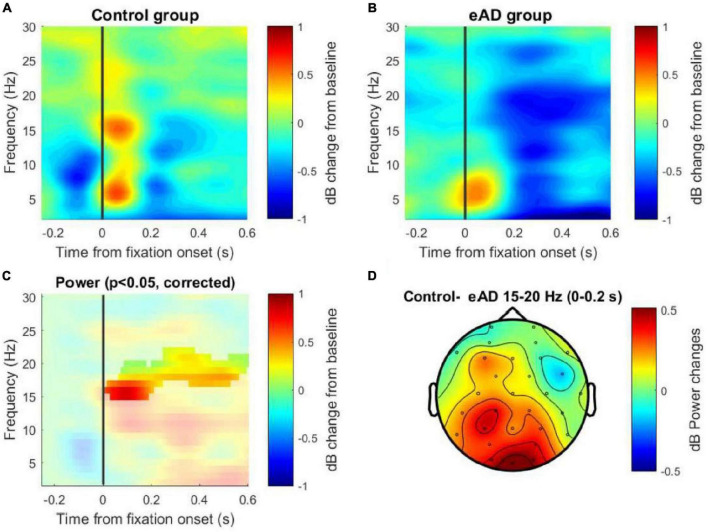
Time-frequency (TF) maps of the Oz channel. **(A)** Power spectral decomposition of the electroencephalography (EEG) data for the control group and **(B)** for the early Alzheimer’s disease (eAD) group. **(C)** Cluster-based permutation test on TF activity of the Oz channel. The analysis considered 1,000 permutations to determine the cut-off alpha = 0.025, to two tails, resulting in significant differences in the beta band between 15 and 20 Hz. **(D)** Topographical map from the differences in power data averaged. The solid line (vertical line) represents the zero time at the beginning of the eye fixation. The color scale represents the percentage of change relative to the baseline period of –750 to –450 ms and normalized in decibels.

We performed a power spectra analysis to identify differences in the frequency band. The TF decomposition was achieved by generating epochs in the signal associated with the eye fixation and taking a baseline of −750 to −450 ms. Two examples of TF maps for each group are shown in Supplementary Figure 3. Both the control and the eAD group showed a marked predominance of activity for the Oz channel in the low-frequency spectrum, particularly in theta and alpha bands, but also an activity relevant in the beta band that went only recognized in the control group (Figures 4A, B).

A two-tailed non-parametric cluster-based permutation test for the Oz channel was performed to test for differences in the power between the groups. This test considered the TF data from −250 ms to 600 ms, which means that we include the window to involve the visual event and its early cognitive processing, and this is for a frequency range that considers low frequencies 2–25 Hz. A Monte Carlo estimate of the permutation *p*-value was computed by randomly permuting condition labels (*N* = 1,000). We observed significant differences in the beta band between 15 and 18 Hz for the first 250 ms post-fixation and the same effect between 17 and 20 Hz for the 250– 600 ms post-fixation (Figure 4C). Additionally, the power difference (control–eAD) is shown in a topographic map for the beta frequency band, within the range of 15–20 Hz in the time interval between 0 and 200 ms (Figure 4D). This difference could be associated with difficulties in spatial navigation when the reactivation of memories is necessary to find the platform using visual cues.

### Functional connectivity analysis

**FIGURE 5:**
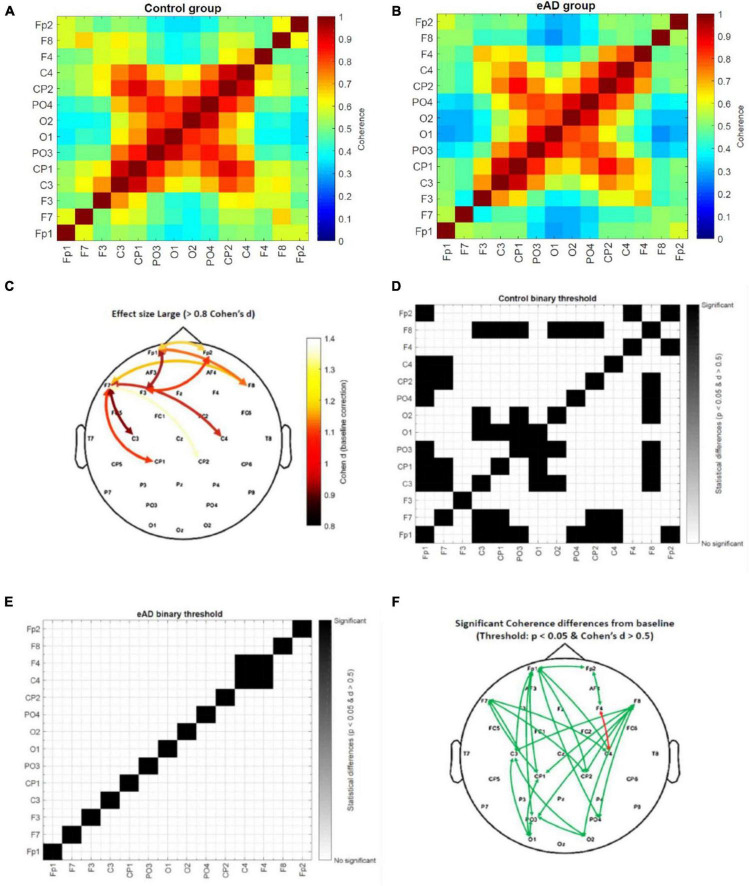
Coherence beta (15–20 Hz) connectivity matrices from 0 to 300 ms. **(A)** Absolute coherence matrix for the control group, and **(B)** early Alzheimer’s disease (eAD) group. The color scale represents low coherence in blue and high coherence in red between a pair of electrodes. **(C)** Topographic distribution of electrode pairs with large value effect size (>0.8 Cohen’s *d* statistic). Threshold value matrix. **(D)** Matrix of control group and **(E)** matrix of eAD group. The color scale represents significant coherence differences for the baseline time (–750 to –450 ms) in black. In white, there is no difference in baseline time for the activity of the electrode pair, using as a threshold the *p* < 0.05 and Cohen’s *d* statistic > 0.5. **(F)** Topographic distribution of electrode pairs with significant coherence differences from baseline time by groups. The green arrow represents the control group, and the red arrow the eAD group.

We applied a pairwise electrode coherence analysis for the eAD and control groups in the frequency beta-band. These pairwise electrodes were principally related to the frontoparietal activity, whose functions are widely associated with working memory and are particularly relevant to visual working memory and visual attention. Evidence suggests that the activity of this component correlates with visual processing. The mean coherence between 14 channels of eAD and the control group in the beta frequency (15–20 Hz) showed in Figures 5A, B. We calculated the connectivity matrices with the baseline correction from −750 to −450 ms and the *p*-value matrix (one-sample *t*-test between groups). We found significant uncorrected differences in coherence distributed in frontal and frontoparietal areas for the beta band. We estimated the effect size (Cohen’s *d*) for the coherence spectrum in the frontoparietal-occipital axis to understand the impact of these results (Supplementary Figures 4A–D).

Only the high values of Cohen’s *d* were used in a topographic map highlighting which areas could impact the participants’ behavioral performance (Figure 5C). We found that most of the changes in eAD coherence were essentially frontoparietal and that less activation or synchronization in the prefrontal cortices will account for poorer spatial navigation. In addition, we created a binary connectivity matrix, where the threshold represents a combination of previously calculated values in the beta frequency band; an effect size (>0.5), and the *P*-value matrix for the one-sample *t*-test (<0.05). The scale represents significant coherence differences from the baseline in black color. The statistical analysis results of the intragroup coherence for the control connectivity matrix show many pairwise comparisons with significant differences (Figure 5D). The eAD matrix shows only one electrode pair with significant brain activity changes (Figure 5E). Finally, we show the topographic distribution of electrode pairs with significant coherence differences from baseline time by groups (Figure 5F). Based on these results, we suggest that long- and short-distance synchrony exhibits a higher degree of coherence differences in the frontoparietal region of the brain. That could affect less effective planning of navigation routes since this region is critical for executive functions, including planning, organization, error monitoring, and decision-making.

## Discussion

Despite the vast progress in recent years in the study of AD, the principal mechanisms involved in cognitive deficits still need to be clarified. This study compared behavioral performance, ocular behavior, and functional connectivity in control vs. eAD subjects in a sensitive early detection test of cognitive impairment (Laczó et al., 2011; Weniger et al., 2011; Gazova et al., 2012, 2013; Tarnanas et al., 2015). Our results showed significant differences between groups in the prefrontal area in beta-band coherence, a consequence of less effective planning of navigation strategies.

### Early Alzheimer’s disease and spatial navigation in the virtual maze

Patients with a diagnosis of eAD performed significantly worse in the VMWN task than the control group, as they did also in all the behavioral aspects evaluated in this work. Although differences in speed were observed between the two groups, we attribute the worse performance of AD patients in the spatial navigation task to memory problems and how they use visual cues to find the platform. Performance of AD participants had more erratic trajectories and longer reaction times before initiating spatial navigation strategies in AD participants. Moreover, the results presented in Figure 1G show that their latency to find the platform after it became visible was not different from control subjects, which supports the idea that it is not a locomotor problem for the AD subjects managing to execute basic motor navigation programs. This lower latency time could be related to the increased speed of response in searching the platform. These results reaffirm that a deficit in space coding used for navigation learning is an early disease feature. In support of our findings, recent studies have used virtual tools to indicate that the performance of participants at high risk of developing AD is worse than that of the control groups (Gazova et al., 2012; Laczó et al., 2012; Tarnanas et al., 2012, 2015; Vlček and Laczó, 2014), as is reported in a study that has patients walk through a real maze (Benke et al., 2014). Our results strongly suggest that a virtual task based on spatial labyrinths may constitute a practical and sensitive tool for eAD detection.

Analyzing the different aspects recorded during navigation, eAD participants displayed some well-marked characteristics: (i) lower orientation capacity, estimated from a higher latency in finding the platform, higher error rates, and lower travel speeds. (ii) Lower capacity for spatial learning, evidenced by the lack of an improvement in the error rate throughout the repetitions of the task, a capability observed in the control group. (iii) Reduced avidity to find the platform, manifested as shorter average run lengths and slower speed, elicited after the platform appeared in the scene. In other studies comparing healthy-elderly people to participants with MCI, these features were also detected (Perrochon and Kemoun, 2014; Vlček and Laczó, 2014; Tarnanas et al., 2015).

It is worth noting that evidence indicates gender differences in spatial memory. Men perform better than women in spatial navigation tasks, and they also travel longer distances without making course changes, pause less often, and return less to previously visited places (Munion et al., 2019). Differences in performance in navigation tasks could be explained by the fact that they produce different way finding behaviors. Nevertheless, considering that AD affects both men and women and that our data have shown no significant differences between the number of them in the groups, we decided to analyze the overall group performance.

### Ocular behavior in early Alzheimer’s disease

Early AD patients performed significantly different exploration strategies to reach the visual keys in the task. These findings reassert an increased instability in fixations, enhanced latency of voluntary exits, the erratic direction of microsaccades, a higher number of anti-saccadic errors, and decreased correction of this anti-saccadic error in AD participants (Anderson and MacAskill, 2013; Kapoula et al., 2014). The eye fixations of the control group focused on task-relevant objects (visual keys and possible platform location). Instead, in the eAD group, the visual exploration was more heterogeneous on the image and without a clear preference for regions of interest that could guide solving the task. All these deficits might represent an early manifestation of AD impairments in executive functions and visual parameters such as visuospatial skills, processing, and selective visual attention (Chehrehnegar et al., 2020). Furthermore, the participants with eAD had more eye fixations over the middle line and less on the bottom relative to controls. This difference can be due to attention deficiencies and the involvement of prefrontal networks and visual attention (Belleville et al., 2008).

### Early electrophysiological features of spatial navigation in Alzheimer’s disease

We found a significantly lower amplitude of the P100 component in eAD participants. These results differ from other studies measuring classical evoked potentials (not fERP), in which there are usually no differences in such components as the P100 (Quiroz et al., 2011). Our results also differ from another study that did not detect a decrease in the P100 amplitude in very mild AD. However, the participants in that study did not have any executive function requirements (Cheng and Pai, 2010). In another study, a virtual exploration model showed significant differences in late components of the classical ERP in patients with amnestic cognitive impairment associated with non-visual events (Tarnanas et al., 2015). The appearance of such early differences in the ERP associated with fixation is difficult to attribute to the learning of the task at the hippocampus level but instead to an altered visual-executive system of the participants with eAD that prevents their optimal processing in the early stages of visual processing. This finding has implications for the entorhinal cortex and hippocampal activity and, thus, for the consolidation and retrieval of sensory information. In agreement with this hypothesis, a study that evaluated ERP variables in a visual exercise demonstrated that those AD participants who had disturbances in the resting state, in turn, presented lower amplitudes of ERP (Tartaglione et al., 2012). There are, therefore, detectable electrophysiological features recorded with surface EEG during space exploration in participants with eAD.

### Prefrontal beta-band coherence and its role in eAD

Our spectral power analysis through the TF maps presented differences in activity in the theta frequency ranges, alpha, and significantly in the beta band (15–20 Hz), confirmed by the analysis of cluster-based permutations tests. These results are consistent with a study that reported increases in the theta band and decreases in the alpha and beta-band in AD patients (Aghajani et al., 2013). Other studies even attributed the reduction in the alpha band and reduced beta power in the parietal and occipital regions as a differentiating factor between normal aging, MCI, and AD (Jeong, 2004; Kwak, 2006; Rossini et al., 2006; Babiloni et al., 2011; Ruiz-Gómez et al., 2018; Li et al., 2019). The beta-band was related to memory processes in our study, and their decrease in the eAD group could be associated with difficulties in spatial navigation when the reactivation of memories is necessary to find the platform by visual cues (Guran et al., 2019).

Although a single process could not entirely explain beta oscillations, its role in the interneural communication of inhibitory networks and high executive demands has been ascribed (Guevara et al., 2018; Hou et al., 2018). Synchronization, instead, might be involved in sensory processing (Singer, 1993; Hou et al., 2018). Our results showed a reduction in beta band functional connectivity in the eAD group in the prefrontal cortices. Both alpha and beta frequencies are associated with many functions representing downstream influences. However, beta oscillations are crucial for long-distance communication between cortical regions, maintaining a constant update of the state of the brain (Guran et al., 2019).

Our results, as well as previous studies, showed that selective visual attention is sensitive in eAD. Additionally, the prefrontal cortex (PFC) could be involved in associative learning in navigation and changing strategies and might be engaged in processes to search for specific objectives, such as selecting the best route or trajectories in space navigation processes (Zhong and Moffat, 2018). Besides, disconnections between brain regions playing an essential role in cognitive impairment in AD, with reduced synchrony as a marker, were described (Delbeuck et al., 2007; Wang et al., 2014; Engels et al., 2016; Tait et al., 2019). Some studies have proposed activating the right PFC during spatial working memory (WM) tasks in young adults. In contrast, older adults presented bilateral activation of PFC as a compensatory response to cognitive impairment. Likewise, healthy older adults also showed bilateral hyperactivation of the frontal cortex during a particular memory task (such as coding complex visual scenes). During the progression from MCI to AD, it has been proposed that the disintegration of compensatory networks occurs due to the lack of activity observed in the lateral regions of the prefrontal cortex (PFC), precuneus, and posterior parietal cortex. These regions are all involved in the executive function compared to controls (Clément et al., 2013; Kirova et al., 2015). These findings are consistent with our proposal that the symptoms observed in patients with eAD are closely related to a loss of functional connectivity, reflected by physiological changes and an attenuation of the electrical activity (Babiloni et al., 2010; Wang et al., 2014; Engels et al., 2016; Blinowska et al., 2017).

It is interesting to note that recent studies have shown that the brain undergoes significant structural and functional changes at older ages, and neurodegenerative processes can accelerate these changes. Many of these functional changes are found in the PFC as a compensatory response to the processes of cognitive scaffolding, enhancing activity in these areas *via* the recruitment of additional regions or networks (Ferreira and Busatto, 2013; Sala-Llonch et al., 2015). This occipitofrontal desynchronization in subjects with AD is part of this compensatory mechanism rather than just a poor visual-spatial ability during navigation tasks. Although our results are task-dependent, we suppose that with additional resting-state studies, it is possible to show similar patterns of functional brain connectivity in subjects with AD.

As we conjectured, spatial navigation impairments can be associated with early mechanisms of cognitive deterioration in the progression to AD. This task joints the functions of the occipital, parietal, and frontal cortices. We already know that navigation abilities depend on the occipital cortices for the early processing of visual information and the parietal and hippocampal cortices for the generation of the cognitive map and egocentric/allocentric navigation strategies. At the same time, the frontal cortex is relevant for deciding, developing, and planning actions (Vann et al., 2009). A study by Coughlan et al. (2018) showed that morphological changes (in particular, the accumulation of Aβ in the cerebral cortex) follow a typical course of progression, which coincides with the structures involved in spatial navigation. Thus, the spatial navigation impairments are consistent with the loss of cognitive skills and less functional connectivity in eAD (Pai and Jacobs, 2004; Klimkowicz-Mrowiec et al., 2008; Hamilton et al., 2009; Etchamendy et al., 2012; Alberdi et al., 2016; Tait et al., 2019). In summary, we propose an operational model whose functionality depends on the structural integrity but further on the functional connectivity generated.

## Data availability statement

The raw data supporting the conclusions of this article will be made available by the authors, without undue reservation.

## Ethics statement

The studies involving human participants were reviewed and approved by the Clinical Hospital of the Universidad de Chile, Protocol number: 26/2015. The patients/participants provided their written informed consent to participate in this study.

## Author contributions

IP-R, EB, AP-L, and PM: conceptualization and funding acquisition. IP-R, EB, and PM: methodology. IP-R, RM-S, and SM: formal analysis. IP-R, RM-S, SM, EB, MB, AP-L, and PM: investigation and writing—review and editing. IP-R: writing—original draft and visualization. PM: supervision. All authors read and approved the final version of the manuscript.

## Footnotes

1
www.biosemi.com
