# Supplementary material for: Visual-spatial processing impairment in the occipital-frontal connectivity network at early stages of Alzheimer’s disease
Source: Front Aging Neurosci. 2023 Feb 9;15:1097577. doi: 10.3389/fnagi.2023.1097577 (PMC9947357; doi:10.3389/fnagi.2023.1097577)
Supplement: Supplementary file 1 [file Image_1.pdf]

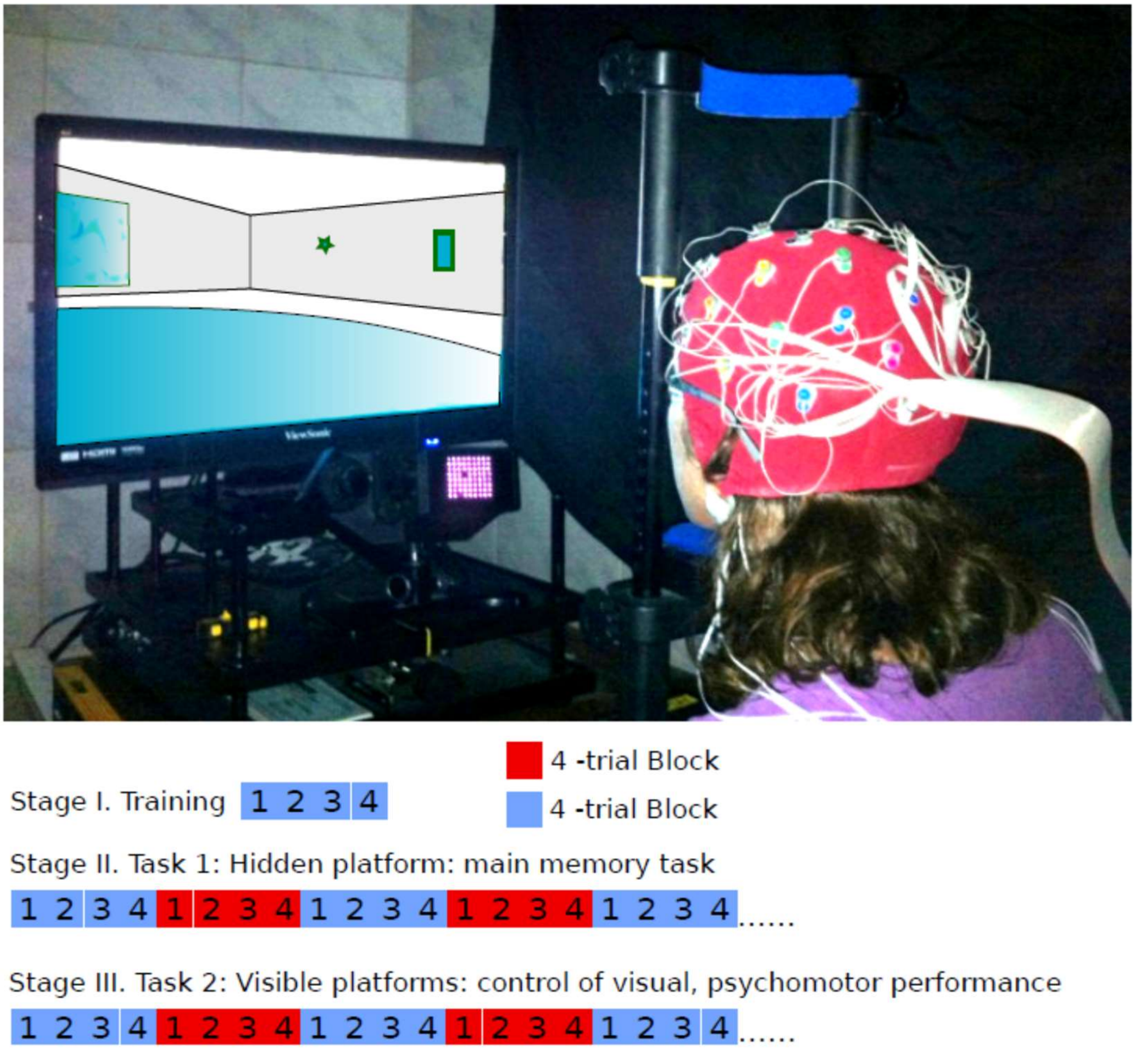

Supplementary Figure 1. Experimental protocol. The upper image shows a typical setup during a recording session using Electroencephalographic (EEG) and eye-tracking systems, applied in this case to a non-experimental subject. Participants navigated using the arrow keys of a standard computer keyboard. The lower scheme represents the sequence of epochs composing each stage of the experimental protocol: Stage I: Training task; Stage II: Navigation with a hidden platform and Stage III, Navigation with a visible platform. Two minutes breaks (red) were interspersed between each block of four trials (blue).
