# Supplementary material for: Visual-spatial processing impairment in the occipital-frontal connectivity network at early stages of Alzheimer’s disease
Source: Front Aging Neurosci. 2023 Feb 9;15:1097577. doi: 10.3389/fnagi.2023.1097577 (PMC9947357; doi:10.3389/fnagi.2023.1097577)
Supplement: Supplementary file 2 [file Image_2.pdf]

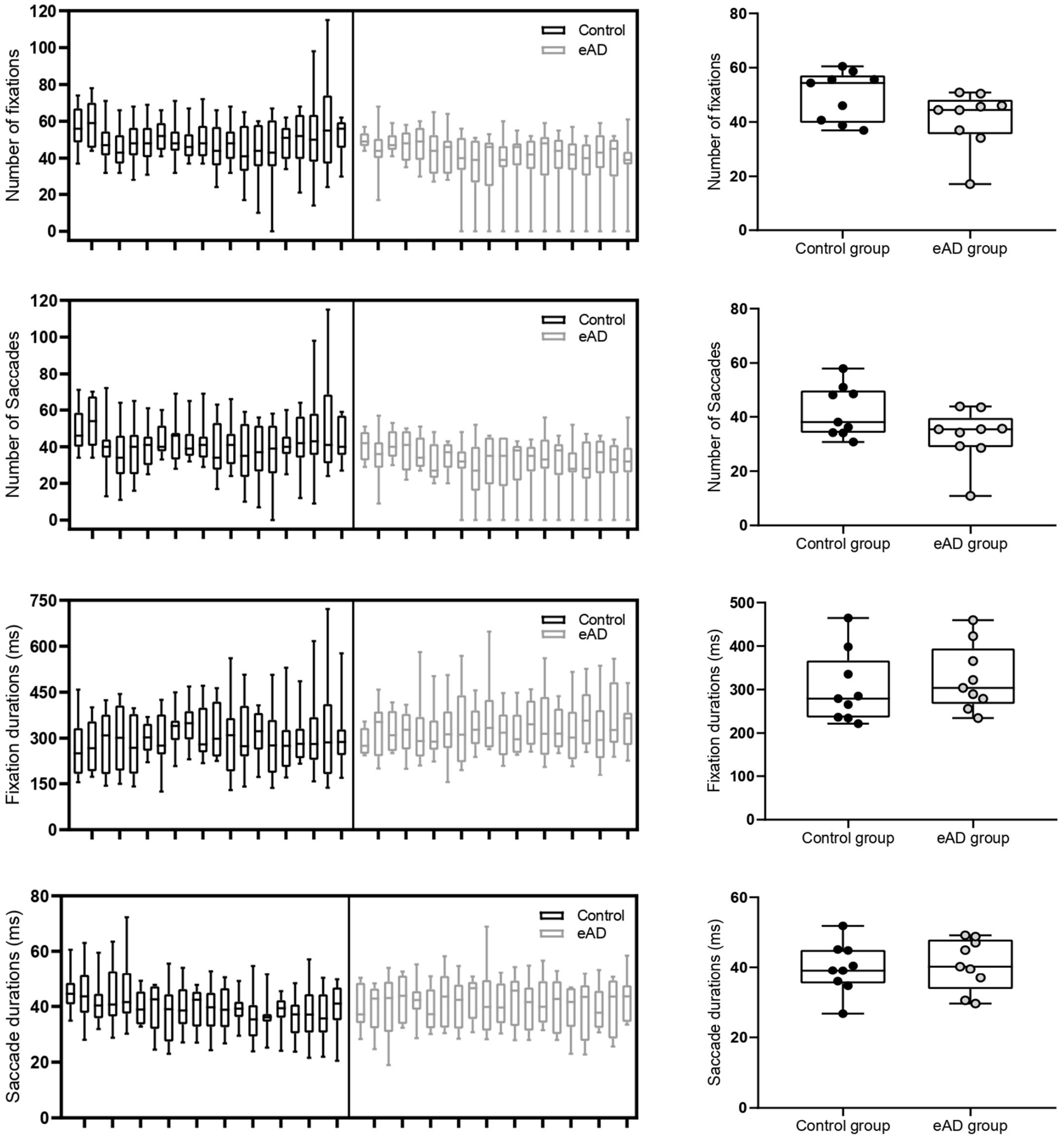

Supplementary Figure 2. Comparison of ocular behavior between the eAD and control participants. From upper to lower, the rows represent: number of fixations, number of saccades, fixation duration (ms), and saccade duration (ms). Nested graphs (left) show the basic ocular behavior parameters quantified for both, eAD and control groups. Box plots for the mean of the number and duration of ocular parameters (fixation-saccades) per trial. The Box-whisker plots at the right for eAD and control groups represent the median of the number and duration of ocular movements of 9 participants per 20 trials for each group. Statistical significance was assessed by the Mann-Whitney test (*$p < 0.05$). Gray: eAD participants (n=9); Black: HC participants (n=9).
