# Supplementary material for: Visual-spatial processing impairment in the occipital-frontal connectivity network at early stages of Alzheimer’s disease
Source: Front Aging Neurosci. 2023 Feb 9;15:1097577. doi: 10.3389/fnagi.2023.1097577 (PMC9947357; doi:10.3389/fnagi.2023.1097577)
Supplement: Supplementary file 3 [file Image_3.pdf]

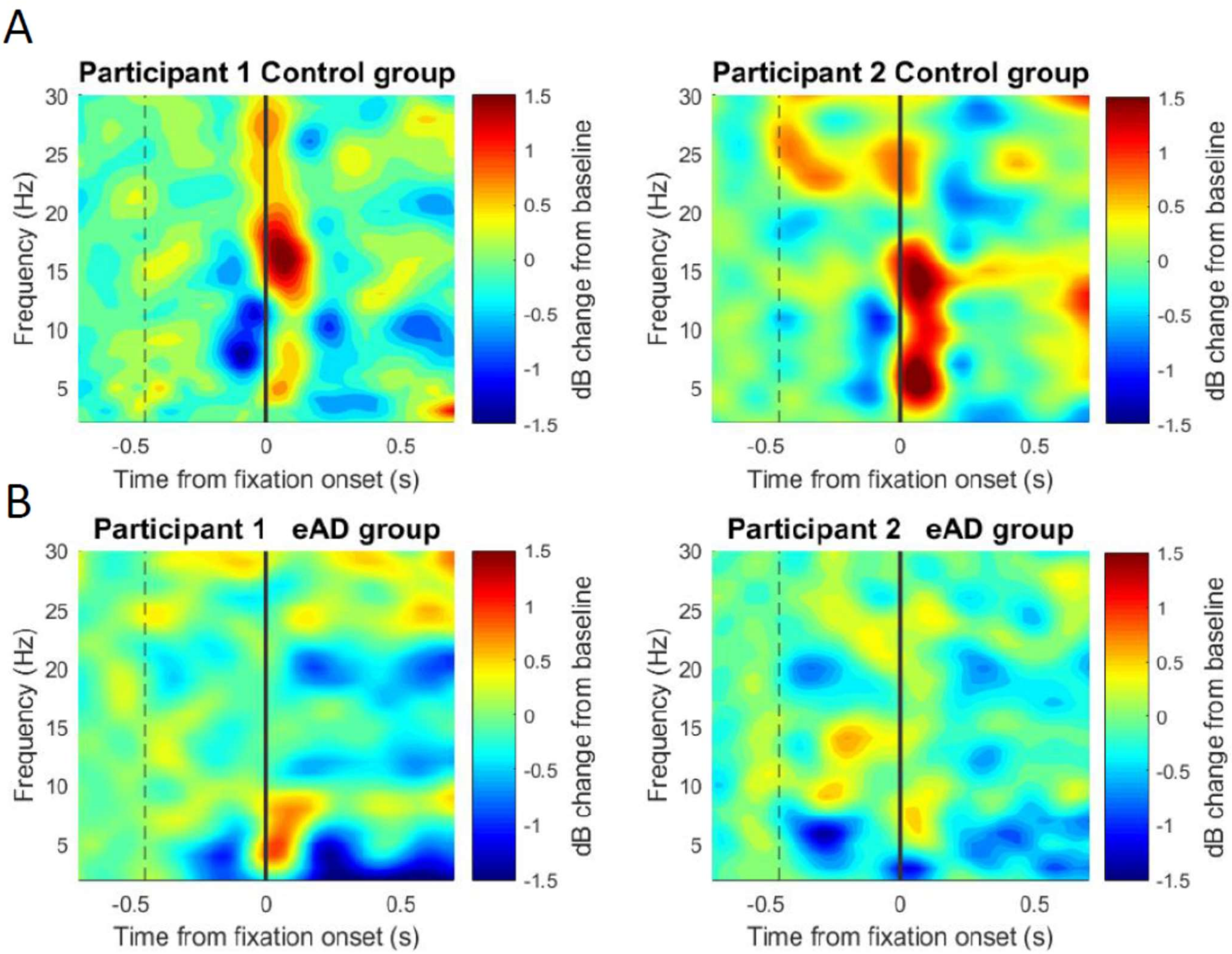

Supplementary Figure 3. Power spectrogram of the Oz channel. a) Time-frequency power spectral decomposition of the EEG data of two participants from the Control group at the Oz electrode, b) Time-frequency power spectral decomposition of the EEG data of two participants from the eAD group at the Oz electrode. The solid line (vertical line) represents the zero time at the beginning of the eye fixation, and the dashed line indicates the start of the baseline. The color scale represents the percentage of change relative to the baseline period of -750 to -450 ms and normalized in decibels.
