# Supplementary material for: Visual-spatial processing impairment in the occipital-frontal connectivity network at early stages of Alzheimer’s disease
Source: Front Aging Neurosci. 2023 Feb 9;15:1097577. doi: 10.3389/fnagi.2023.1097577 (PMC9947357; doi:10.3389/fnagi.2023.1097577)
Supplement: Supplementary file 4 [file Image_4.pdf]

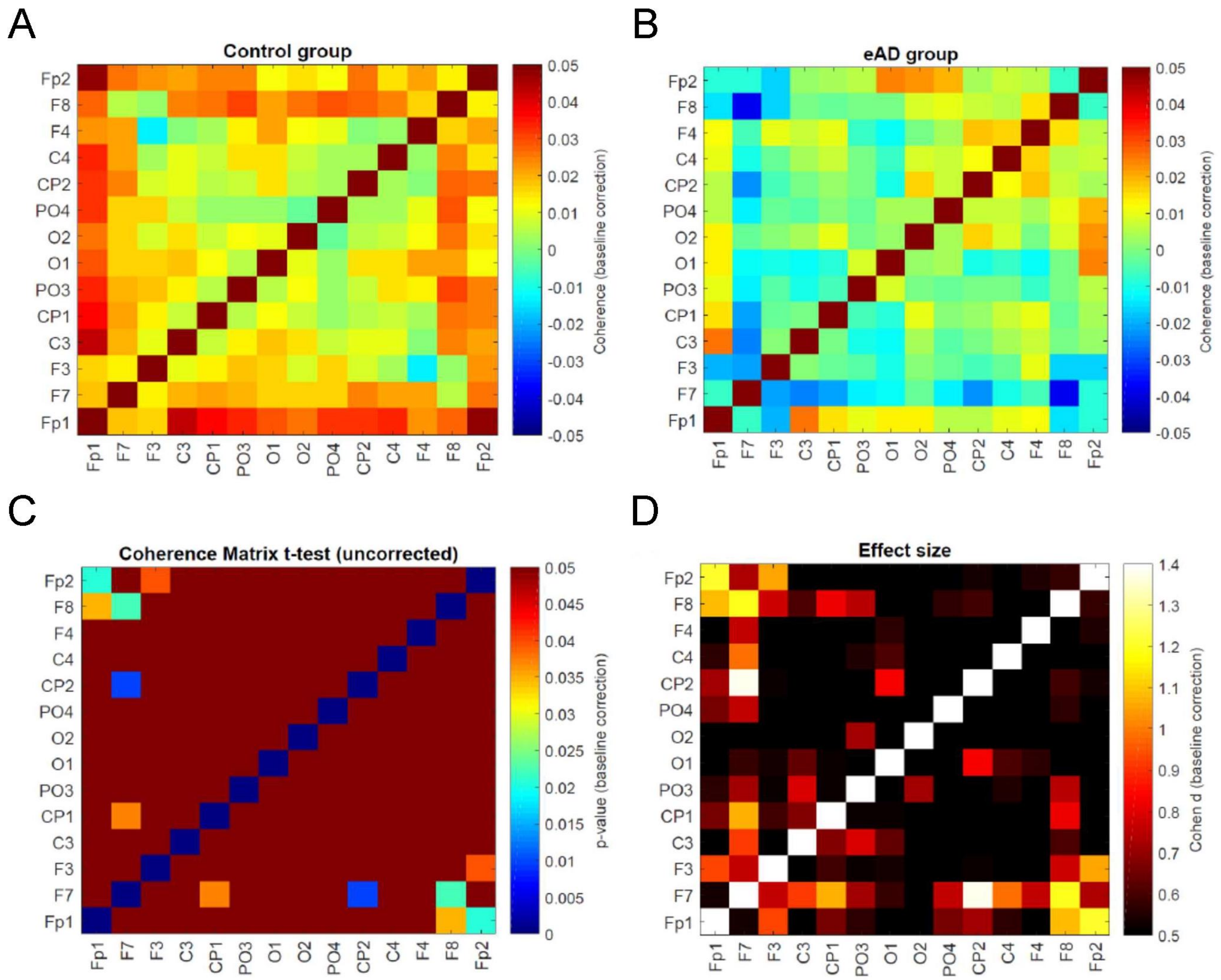

Supplementary Figure 4. Coherence Beta (15-20 Hz) connectivity matrices from 0 to 300 ms. Connectivity matrices with baseline correction from -750 to -450 ms A) Coherence matrix for the control group, B) Coherence matrix for the eAD group. The color scale represents low coherence in blue and high coherence in red between the pair of electrodes. C) Matrix of P-value of the two groups obtained by t-test, the highest value with red color, and D) Matrix of differences between groups for an effect size > 0.5 Cohen's d statistic (medium-large effect size) Color scale represents in black (dark color) medium differences of effect size and in white (light color) marked differences of effect size for the pair of electrodes.
