# Supplementary material for: Visual-spatial processing impairment in the occipital-frontal connectivity network at early stages of Alzheimer’s disease
Source: Front Aging Neurosci. 2023 Feb 9;15:1097577. doi: 10.3389/fnagi.2023.1097577 (PMC9947357; doi:10.3389/fnagi.2023.1097577)
Supplement: Supplementary file 5 [file Table_1.docx]

**Demographic and clinical characteristics of the participants.**

| **Characteristic** | **MCI**  number (%)  or mean ± SD | **Control**  Number (%)  or mean ± SD | **p value** |
| --- | --- | --- | --- |
| ***Sample*** |  |  |  |
| Size | 9 | 9 |  |
| Age | 76.67 ± 6.16 | 71.22 ± 8.48 | 0.2138 |
| Range (min-max) | 69-88 | 61-84 |  |
|  |  |  |  |
| ***Gender*** |  |  | 0.6199 |
| Male | 2 (22.22%) | 4 (44.44%) |  |
| Female | 7 (77.78%) | 5 (55.56%) |  |
|  |  |  |  |
| ***Education (years)*** | 12.33 ± 4.03 | 16.89 ± 4.31 | 0.0299 |
|  |  |  |  |
| ***Neuropsychological measures*** |  |  |  |
| CDR-SOB scores | 0.89 ± 0.22 | 0 | < 0.001 |
| MoCA scores | 20.44 ± 3.32 | 29.22 ± 0.83 | < 0.001 |
| MoCA-MIS | 9.56 ± 2.19 | 14.78 ± 0.44 | < 0.001 |
| MMSE scores | 23.22 ± 2.05 | 29.78 ± 0.44 | < 0.001 |

**Supplementary Table 1.** Continuous variables are reported as mean ± standard deviation (SD), while categorical variables are expressed as frequencies (%). Wilcoxon rank-sum test was used for age, education, CDR-SOB, MoCA, MoCA-MIS, and MMSE comparison between groups. A chi-square test was used for gender comparison (Fisher's exact test). HC, Healthy controls; veAD, very early Alzheimer's Disease; CDR-SOB, Clinical Dementia Rating Scale Sum-of-Boxes; MoCA, Montreal Cognitive Assessment; MoCA-MIS, Montreal Cognitive Assessment Memory Index Score; and MMSE, Mini-mental State Examination.
